# Hydroxychloroquine and short-course radiotherapy in elderly patients with newly diagnosed high-grade glioma: a randomized phase II trial

**DOI:** 10.1093/noajnl/vdaa046

**Published:** 2020-04-27

**Authors:** Lucy Brazil, Angela L Swampillai, Ka Man Mak, Darren Edwards, Pavlina Mesiri, Laura Clifton-Hadley, Richard Shaffer, Joanne Lewis, Colin Watts, Sarah Jeffries, Pinelopi Gkogkou, Anthony J Chalmers, Naomi L Fersht, Allan Hackshaw, Susan C Short

**Affiliations:** 1 Guy’s and St Thomas’ NHS Foundation Trust, London, UK; 2 Cancer Research UK and UCL Cancer Trials Centre, London, UK; 3 ClinBAY Ltd., Limassol, Cyprus; 4 Royal Surrey County Hospital, Guildford, UK; 5 Northern Centre for Cancer Care, Freeman Hospital, Newcastle Upon Tyne Hospitals Trust, Newcastle, UK; 6 University of Birmingham/Queen Elizabeth Hospital, Birmingham, UK; 7 Cambridge University Hospitals NHS Foundation Trust, Cambridge, UK; 8 Norfolk and Norwich University Hospitals, Norwich, UK; 9 University of Glasgow, Glasgow, UK; 10 UCLH NHS Foundation Trust, London, UK; 11 St James’s University Hospital, Leeds, UK

**Keywords:** de novo, glioma, hydroxychloroquine, radiotherapy, trial

## Abstract

**Background:**

Effective treatment for patients at least 70 years with newly diagnosed glioblastoma remains challenging and alternatives to conventional cytotoxics are appealing. Autophagy inhibition has shown promising efficacy and safety in small studies of glioblastoma and other cancers.

**Methods:**

We conducted a randomized phase II trial to compare radiotherapy with or without hydroxychloroquine (2:1 allocation). Patients aged at least 70 years with newly diagnosed high-grade glioma deemed suitable for short-course radiotherapy with an ECOG performance status of 0–1 were included. Radiotherapy treatment consisted of 30 Gy, delivered as 6 fractions given over 2 weeks (5 Gy per fraction). Hydroxychloroquine was given as 200 mg orally b.d. from 7 days prior to radiotherapy until disease progression. The primary endpoint was 1-year overall survival (OS). Secondary endpoints included progression-free survival (PFS), quality of life, and toxicity.

**Results:**

Fifty-four patients with a median age of 75 were randomized between May 2013 and October 2016. The trial was stopped early in 2016. One-year OS was 20.3% (95% confidence interval [CI] 8.2–36.0) hydroxychloroquine group, and 41.2% (95% CI 18.6–62.6) radiotherapy alone, with a median survival of 7.9 and 11.5 months, respectively. The corresponding 6-month PFS was 35.3% (95% CI 19.3–51.7) and 29.4% (95% CI 10.7–51.1). The outcome in the control arm was better than expected and the excess of deaths in the hydroxychloroquine group appeared unrelated to cancer. There were more grade 3–5 events in the hydroxychloroquine group (60.0%) versus radiotherapy alone (38.9%) without any clear common causation.

**Conclusions:**

Hydroxychloroquine with short-course radiotherapy did not improve survival compared to radiotherapy alone in elderly patients with glioblastoma.

**Key Points**

- Hydroxychloroquine plus short-course radiotherapy (RT) did not improve survival compared to RT.
- Standard doses of hydroxychloroquine may not enhance tumor regression with short-course RT.

**Importance of the Study**

This study represents the largest of 3 randomized trials in glioma and hydroxychloroquine (HCQ)/chloroquine. About 54 patients were randomized to receive radiotherapy with or without HCQ (2:1 allocation). The trial was stopped early in 2016 as the outcome in the control arm was better than expected and the excess of deaths in the HCQ group appeared unrelated to cancer. The results add useful information to the literature regarding the potential value of autophagy inhibition in glioma and other solid tumors. Our findings suggest that standard doses of hydroxychloroquine might not produce enhanced tumor regression with short-course radiotherapy. Investigators of similar trials being conducted should also carefully monitor progression-free survival and overall survival. Results from ongoing and planned studies in glioma as well as other malignancies will further contribute to the growing evidence base for this type of therapy.

The incidence of glioblastoma (GBM) appears to be increasing.^1,2^ In the UK, for example, the age-standardized rate has doubled to 5 per 100 000 person-years from 1995 to 2015, including in elderly patients.^1^ These gliomas are especially difficult to treat, with a median survival of less than 12 months. Treatment options include debulking surgery, temozolomide chemotherapy, radiotherapy, and combinations of these. However, conventional chemo-radiotherapy delivered over 6 weeks is often poorly tolerated in patients aged older than 70 and can lead to worse outcomes than less intensive regimens.^3–5^ Short-course radiotherapy with temozolomide in this patient group seems effective, though the benefit of adding temozolomide might be more pronounced for tumors exhibiting *O*^6^-methylguanine-DNA-methyltransferase (MGMT) promoter methylation (about 40%).^6–8^ Although shorter treatment is tolerable for elderly patients and appealing because of fewer hospital visits, alternatives to conventional cytotoxics are needed.

Autophagy has been proposed as a relevant aspect of cancer biology for many years, although its role in either tumor promotion or prevention is debated.^9^ Increased autophagic flux allows tumor cell growth and survival, and conversely, autophagy can prevent tumor proliferation and inflammation.^10,11^ Either enhancing or inhibiting autophagy appears to have positive therapeutic effects, but most research is focused on inhibition.^10^ Preclinical and small early-phase clinical studies have investigated the autophagy inhibitors hydroxychloroquine or chloroquine for various cancers but with mixed findings.^12–14^ In GBM, interest has been in using these agents as a radiation sensitizer and as adjuvant treatment, but their tumor-suppressive properties are not fully understood, and inhibition alone might be insufficient to alter outcomes.^15^

Two small clinical trials and a retrospective review of patient records conducted before 2007 (all from the same institution) showed promising results in GBM. In a double-blind, placebo-controlled trial, patients aged younger than 60 years received standard chemo-radiotherapy with or without 12 months of chloroquine. The median overall survival (OS) was 24 months for those given chloroquine but 11 months with placebo (hazard ratio [HR] 0.52, *P* = .14).^16^ A non-blinded randomized trial showed a marked increase in survival (median 33 months with chloroquine vs 11 months without, *P* < .001).^17^ Finally, among 41 GBM patients who chose to have chloroquine as part of their care the median OS was 25 months, compared to 11 months in 82 patients who did not take chloroquine (*P* < .001).^18^

This prior evidence led us to develop a randomized study to examine the efficacy and tolerability of hydroxychloroquine combined with short-course radiotherapy, specifically in elderly patients. We used hydroxychloroquine instead of chloroquine because it has a better toxicity profile.

## Patients and Methods

We conducted an open-label randomized phase II trial.

### Patients

Patients were eligible if they were aged at least 70 years with newly diagnosed histologically confirmed high-grade glioma, ECOG performance status 0/1, and life expectancy of more than 2 months. They had to have adequate biochemistry, blood counts, and a Mini-Mental State Examination (MMSE) score at least 17 which is indicative of mild or no cognitive impairment. Prior macular degeneration, diabetic retinopathy, concurrent psoriasis, G6PD deficiency, and any serious medical/psychological condition precluding study therapy, other malignancy, or previous therapy for glioma were exclusion criteria. All patients provided written informed consent and the trial was conducted in accordance with the Declaration of Helsinki. The protocol was approved by the national research ethics committee.

### Treatment

Patients were randomized (2:1) to receive short-course radiotherapy with or without hydroxychloroquine (randomization was by minimization using sex and hospital as stratification factors). When the study was designed, temozolomide was not routinely used in this patient group hence MGMT status was not tested or included as a stratification factor.

Radiotherapy was planned to start within 28 days postsurgery (±3 days), delivered as 30 Gy in 6 fractions (5 Gy/day given on 3 nonconsecutive days over 2 weeks). Radiotherapy was planned using 3D-conformal computerized tomography based on pre- and postoperative imaging, with scans taken at 2–3 mm intervals through the brain. Target volume definition was performed according to ICRU-50 and -62 guidance. The following organs at risk were delineated in all patients: optic chiasm, right and left optic nerves, pituitary gland, right and left ocular globes, right and left lenses, brain stem, and spinal cord.

Hydroxychloroquine was given orally as 200 mg tablets, twice daily, starting between 14 and 20 days postsurgery and at least 7 days before commencing radiotherapy. It was continued until progression or unacceptable toxicity including abnormal biochemistry or the development of visual symptoms. No dose modifications were allowed.

Concomitant use of enzyme-inducing antiseizure medications was not permitted. Oral steroid medications were used according to local practice.

### Endpoints and Assessments

The primary endpoint was 1-year OS, measured from the date of randomization to the date of death from any cause. Patients who were alive were censored at the date last seen. Other endpoints included toxicity, progression-free survival (PFS), health-related quality of life (QoL), MMSE, and steroid dependence. PFS was defined as the time between randomization and death from any cause, local tumor progression or recurrence based on clinical evaluation. Patients without a PFS event were censored at the date last seen.

Baseline assessments included postoperative magnetic resonance imaging (MRI) within 20 days of surgery, biochemistry, and ophthalmology screening to exclude macular degeneration. Patients completed QoL questionnaires (EORTC-QLQ-C30 and the brain cancer module BN20) and the MMSE. Follow-up visits occurred at weeks 4, 8, and 12 post-completion of radiotherapy and then bimonthly. Patients on hydroxychloroquine had an electrocardiogram (ECG) and ophthalmology review. The study protocol mandated MRI at the following time points: prior to surgery, following surgery, at 12 weeks following treatment, and thereafter as per standard clinical practice, scans were also undertaken if there was clinical suspicion of progression.

### Statistical Considerations

We used a single-arm phase II A’Hern design, with a target 1-year OS rate of 40% using hydroxychloroquine, assuming 25% for radiotherapy alone (equivalent to a median survival of 9 vs 6 months). With one-sided 15% statistical significance and 80% power, we required 38 patients to be given hydroxychloroquine, of which 13 need to be alive at 1 year to justify further investigation. We also aimed to recruit 19 control patients (to represent a 2:1 randomization allocation ratio), but the trial was not powered for a direct comparison between the groups.

HRs, along with 95% confidence intervals (CI) and two-sided *P*-values, were calculated using Cox proportional hazards regression. The survival curves with the estimated 1 year and 6-month rates were calculated using the Kaplan–Meier method. Adverse events were graded using the National Cancer Institute Common Terminology Criteria for Adverse Events, v4. For each patient, the worst grade for each event type was used. Also, for each patient, the change in QoL score from baseline to 8 or 12 weeks posttreatment was calculated and compared between the treatment arms using the Wilcoxon test. All analyses were performed on an intention-to-treat basis using SAS (version 9.4).

The trial was closed early (October 20, 2016) upon recommendation of the independent data monitoring committee (IDMC), because the data showed that the target efficacy for patients given hydroxychloroquine could not be met.

## Results

### Patient Characteristics

**Figure 1.**
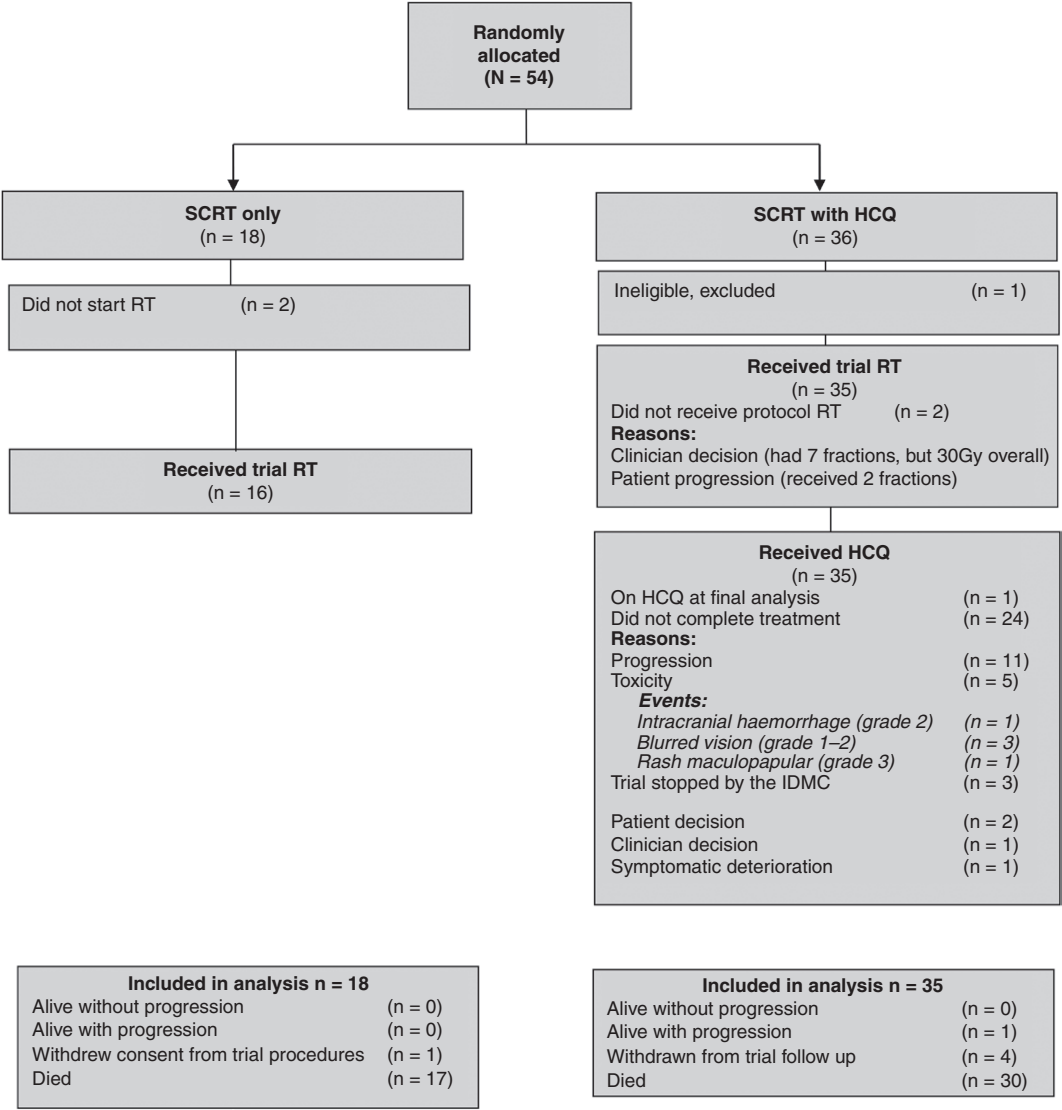
Consort diagram.

Fifty-four patients were randomized between May 2013 and September 2016 from 16 neuro-oncology centers across the UK National Cancer Research Network; one patient was found to be ineligible before starting hydroxychloroquine so was excluded from all analyses (Figure 1). Most patients were male (62%), the median age was 75 (range 70–83), and the majority of cases were diagnosed with GBM (89%). Baseline characteristics (Table 1) were well balanced except for ECOG status in which a chance imbalance led to more patients who had performance status 0 being in the radiotherapy alone arm than the hydroxychloroquine arm: 38.9 versus 11.4%.

**Table 1.** Baseline Characteristics

Baseline Characteristics		SCRT Only (*n* =18)	SCRT + HCQ (*n* = 35)
Sex, *n* (%)	Female	6 (33.3%)	14 (40.0%)
	Male	12 (66.7%)	21 (60.0%)
Histology, *n* (%)	Anaplastic astrocytoma	1 (5.6%)	2 (5.7%)
	Glioblastoma	15 (83.3%)	32 (91.4%)
	Gliosarcoma	1 (5.6%)	0
	High-grade glioma	1 (5.6%)	1 (2.9%)
Surgery, *n* (%)	Biopsy	10 (55.6%)	21 (60.0%)
	Resection	8 (44.4%)	14 (40.0%)
ECOG, *n* (%)	0 (fully active)	7 (38.9%)	4 (11.4%)
	1 (ambulatory but can work)	11 (61.1%)	31 (88.6%)
Age at entry (years)	Median (range)	75.5 (70–82)	74.0 (70–83)
MMSE score	Median (range)	28.5 (17–30)	27.0 (18–30)

SCRT, short-course radiotherapy; HCQ, hydroxychloroquine.

### Radiotherapy and Hydroxychloroquine Adherence

About 51 patients (96%) received 30 Gy radiotherapy, and only 2 patients did not start radiotherapy and 1 patient received only 2 fractions (Figure 1). The median time from surgery to starting radiotherapy was 28 days; the timing was as per protocol for 42 patients, and it was started early (<25 days postsurgery) for 2 patients or started later (>31 days postsurgery) for 5 patients. Patients began hydroxychloroquine 10 days (median) prior to radiotherapy (range 5–21). The median duration of hydroxychloroquine was 94 days (range 11–340).

### Additional Steroid Treatment

Steroid treatment (ie, dexamethasone) had been given at least once during the trial in 17 (94.4%) radiotherapy alone and 34 (97.1%) hydroxychloroquine patients.

### Progression and Survival

When the IDMC decided to stop the trial early, the 1-year OS rate among patients who received hydroxychloroquine was 11% and with 4 more patients to recruit to the hydroxychloroquine group it was clear that the target of 13 being alive at 1 year could not be achieved, and nor could the target OS rate of 40%. Also, the OS HR (hydroxychloroquine vs controls) was 2.18 (95% CI 0.96–4.96, *P* = .06), which was too close to statistical significance to ignore. Although there was no clear reason for the difference in OS it was not considered appropriate to continue to randomize patients to the experimental arm.

A final data chase was undertaken in April 2019 to identify deaths: 47 patients had died, 30 in the hydroxychloroquine arm and 17 in the radiotherapy alone arm. Disease progression was the cause of death in 34 (72%) patients (Supplementary Table 1).

**Figure 2.**
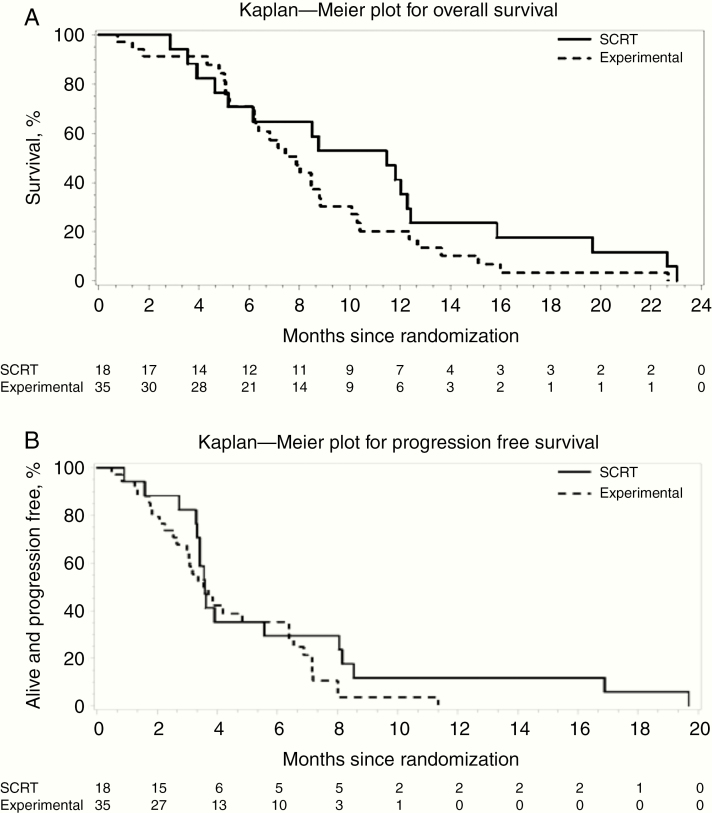
Overall survival and progression-free survival. SCRT: short-course radiotherapy (control arm). Experimental: SCRT plus hydroxychloroquine.

The median OS was 7.9 versus 11.5 months in the hydroxychloroquine and radiotherapy alone arms, with corresponding 1-year OS of 20.3% (95% CI 8.2–36.0) and 41.2% (95% CI 18.6–62.6) (Figure 2). The excess of deaths in the hydroxychloroquine group was partly due to several single events apparently unrelated to cancer (Supplementary Table 1), such as lung infection, pulmonary embolism, and myocardial ischemia. Six patients (5 in short-course radiotherapy arm) had no cause of death recorded.

About 48 patients had a PFS event (progression or death from any cause); 31 in the hydroxychloroquine arm and 17 in the radiotherapy alone arm. The 6-month PFS rate was 35.3% (95% CI 19.3–51.7) with hydroxychloroquine and 29.4 (95% CI 10.7–51.1) in those who received radiotherapy alone (Figure 2).

Although the trial was not designed for a direct comparison, the HR for OS for hydroxychloroquine compared to the control group was 1.55 (95% CI 0.84–2.89, *P* = .16). Because of an apparent imbalance in ECOG status at baseline, we also calculated the HR adjusted for ECOG, which was 1.80 (95% CI 0.82–3.93, *P* = .14).

### Adverse Events

Most patients experienced an adverse event during the trial, as expected for this elderly group with advanced cancer (Table 2). Two fatal adverse events occurred, both in the hydroxychloroquine group (lung infection and myocardial infarction), but neither were considered by the clinician to be related to the trial treatment. Supplementary Table 2 shows the adverse events of special interest in relation to hydroxychloroquine. Eye symptoms were seen in 2 patients (both low grade), and there was no obvious imbalance between the trial groups nor any serious eye problems. There were more grade 3–5 events in the hydroxychloroquine group (60.0%) versus radiotherapy alone (38.9%): 21 versus 7 events (Supplementary Table 3). However, the type of events was heterogeneous. The 5 versus 0 lung infections were noted, but there is no known causal link between hydroxychloroquine and lung infection. Six patients stopped taking hydroxychloroquine early due to adverse events (Supplementary Table 4), with blurred vision as the reason for stopping for 3 patients.

**Table 2.** Adverse Events Summary

Adverse Events	SCRT Only *n* = 18	SCRT + HCQ *n* = 35
	*n* (%)	
Any	15 (83.3)	34 (97.1)
Grade 3–5^a^	7 (38.9)	21 (60.0)
Grade 3–4 of special interest for hydroxychloroquine^a^	2 (11.1)	3 (8.6)
Grade 5 (deaths)	0	2 (5.7)^b^
Discontinued trial treatment due to adverse event^a^	0	6 (17.1)

SCRT, short-course radiotherapy; HCQ, hydroxychloroquine.

^a^See Supplementary Appendix Table 1; Supplementary Tables 2, 3 and 4 for details.

^b^Lung infection and myocardial infarction.

### QoL and MMSE

**Figure 3.**
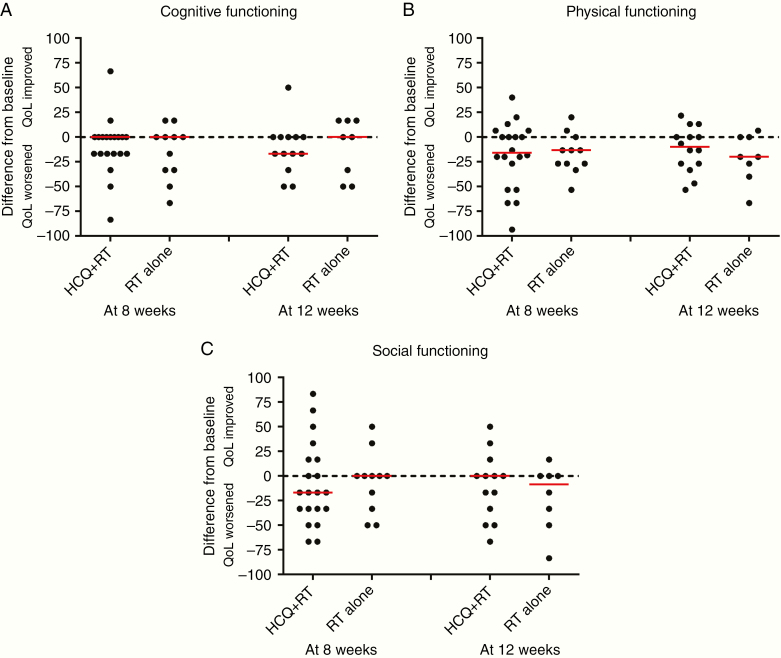
EORTC-QLQC30 quality-of-life functional scales. Scores are on a scale 0 (poor) to 100 (good) health. A positive difference indicates that hydroxychloroquine (HCQ) was better than radiotherapy, and a negative difference indicates that radiotherapy alone was better. The median difference shown by the red bars.

**Figure 4.**
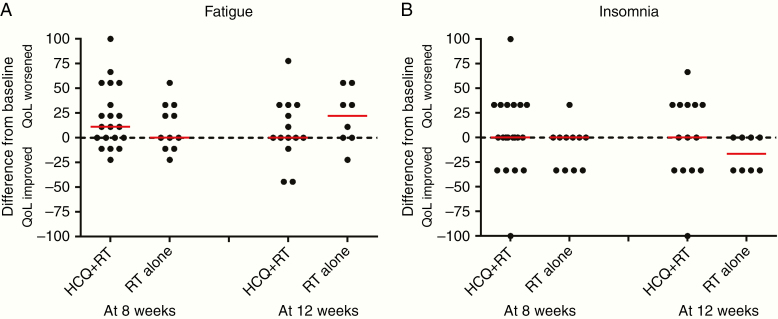
EORTC-QLQC30 quality-of-life symptom scales. Scores are on a scale 0 (good) to 100 (poor) health. A negative difference indicates that HCQ was better than radiotherapy, and a positive difference indicates that radiotherapy alone was better. The median difference shown by the red bars.

Thirty (56.6%) and 22 (41.5%) patients had QoL forms available at both baseline and 8 and 12 weeks, respectively. There were no significant differences in QoL between the treatment arms (Supplementary Table 5; Figures 3 and 4). The median (range) last available MMSE score was 26 (7–29) in the radiotherapy alone and 27 (10–30) in the hydroxychloroquine group (Kruskal Wallis *P* = .38). The median difference (range) in the MMSE score from baseline to the last score was 0 (−15; 0) in the radiotherapy alone and 0 (−14; 4) in the hydroxychloroquine group (Kruskal Wallis *P* = .74).

## Discussion

More than 20% of patients with high-grade glioma are aged at least 70 years when diagnosed. Short-course radiotherapy plus temozolomide is effective,^8^ but is associated with grade 3–4 hematological toxicity (27% lymphopenia, 8% neutropenia, and 11% thrombocytopenia). Elderly patients not eligible for combined chemo-radiation could benefit from either temozolomide alone or hypofractionated radiotherapy alone, with likely greater benefit from temozolomide in patients with MGMT promoter methylated tumors.^19^ At the time this study was designed, prior to reports of the relevance of temozolomide in these patients, MGMT testing was not carried out routinely for this patient group hence it was not used as a biomarker in this study. The strongest effect of MGMT status is in patients treated with temozolomide, which was not part of treatment in our study. in view of this, and the fact that the difference in HR between our groups was large, we do not think it likely that a subgroup effect explains the outcome data. It is noteworthy that there remain very few randomized trials in this patient group, and that to date there has been no formal comparison of the standard chemo-radiotherapy used in younger patients against any alternative regime stratified by MGMT status.

Despite long-running interest in autophagy inhibition for treating cancer and promising data from other groups, our trial did not show improved outcomes for hydroxychloroquine when added to short-course radiotherapy in elderly patients, and OS was lower than in the controls. Short-course radiotherapy alone was effective in our trial patients, exhibiting outcomes better than expected. Although there were only 18 patients in our control group, the median OS (11.5 months) was higher than in the equivalent arm of the trial by Perry et al.^8^ (7.6 months), although the median PFS is similar (3.6 in our study and 3.9 months in the Perry et al. trial). Indeed, our median OS was higher than the patients who received a combination of temozolomide and radiotherapy in that study (9.3 months).^8^ We were reassured that the median OS for our patients who received hydroxychloroquine with radiotherapy (7.9 months) was comparable to the radiotherapy alone arm in the Perry et al. trial (7.6 months), in that adding hydroxychloroquine did not appear to disadvantage patients.^19^ Both trials include similar patients, although there are differences in the radiotherapy regimen and ECOG status. Perry et al. used 40 Gy in 15 fractions delivered over 3 weeks; we used 30 Gy over 6 fractions in 2 weeks. The ECOG status was more favorable in our study, with 21% and 79% who had baseline performance status 0 and 1 respectively, compared to 23%, 54%, and 23% with performance status 0, 1, and 2 in the Perry et al. trial. That trial also included only patients with GBM, whereas we included patients with grade 3 astrocytoma.^19^

Because all patients received radiotherapy in our trial and adherence was high, the noticeable survival difference between the 2 groups could be due to an unexpectedly good outcome in this particular (and small) control group that was a chance finding, rather than a direct detrimental effect of hydroxychloroquine. The better performance status in patients who received radiotherapy only might also influence the difference. Alternatively, there might be some unexplained negative effect of combination therapy. However, the toxicity data do not support this, and the excess causes of deaths in the hydroxychloroquine group did indicate any obvious biologically plausible pattern. Nevertheless, the IDMC felt that they could not ignore the difference in survival, regardless of the reason, so stopped the trial early.

Metabolic reprogramming is a hallmark of many cancers including glioma and metabolic stress is known to activate the autophagy pathway, which is a pro-survival mechanism in this context. Several groups have published data suggesting that inhibition of autophagy can sensitize tumor cells to chemotherapy and radiotherapy.^20,21^ Hydroxychloroquine accumulates in the autophagosome and inhibits the late stages of autophagy that depend on autophagosome and lysosome function. Since it is an extremely well-characterized drug with a low toxicity profile, there are many ongoing studies investigating the additional benefit of hydroxychloroquine in a variety of solid and hematologic malignancies. Experimental studies specifically of glioma cells support the potential for chloroquine to have antitumor activity, on its own or with temozolomide^22–25^; one study showed that it enhances radiation sensitivity.^24^

The 2 small previous randomized clinical trials of GBM which reported remarkable results using chloroquine with radiotherapy were small (18 and 30 patients, respectively) and based on younger patients.^16,17^ However, a later trial of newly diagnosed GBM evaluated hydroxychloroquine combined with radiotherapy and temozolomide: 16 patients in the phase I dose-escalation cohort and 76 in the subsequent phase II stage.^26^ They found that 600 mg per day was the maximum tolerated dose, with no material improvement in OS among the large phase II cohort. Their pharmacokinetic analyses and assessment of autophagy inhibition in peripheral blood lymphocytes led the authors to conclude that autophagy inhibition was not consistently achieved at 600 mg.^26^ Phase I/II studies^27,28^ of mixed advanced solid tumors also suggest that higher doses (1200 mg/day) may be necessary to inhibit autophagy, though in these studies hydroxychloroquine was only combined with either temozolomide or temsirolimus. Although no dose-limiting toxicities were observed, even at the high dose, evidence of antitumor activity mainly occurred in patients with melanoma.^27,28^ These data suggest that the dose we used in our trial (400 mg/day) might have been sub-therapeutic, but when we designed the trial we were mindful of the toxicity evidence at that time in the context of an elderly patient group. There is continued interest in the comparative activity between hydroxychloroquine and chloroquine as well as the use of other chloroquine like agents, including mefloquine, which may have improved blood-brain barrier penetration.^23^

Several other early-phase trials have combined hydroxychloroquine or chloroquine with various established cancer therapies including vorinostat, erlotinib, sirolimus, gemcitabine, and bortezomib with mixed outcomes. The appropriate dose of hydroxychloroquine/chloroquine recommended in these studies has also been variable,^29–34^ partly because of heterogeneity in patient tolerability, which depends on the agent it is combined with. Chloroquine has shown some efficacy in treating brain metastases when added to whole-brain radiotherapy. One trial reported a high local control rate of 88% in 16 patients,^35^ while in a double-blind placebo-controlled trial (73 patients) the one-year PFS rate was 84 versus 55% (chloroquine vs placebo, *P* = 0.008), with a nonstatistically significant improvement in OS (median 10.2 vs 7.4 months).^36^ Ongoing early-phase trials are assessing the effect of adding chloroquine to chemo-radiotherapy in GBM multiforme (NCT023788532, NCT02432417), which may exploit the potential for sensitizing to both radiation and temozolomide.^37^ In one of these (dose-escalation) trials, 13 newly diagnosed patients were given radiotherapy and temozolomide, and the maximum tolerated dose of chloroquine was determined to be 200 mg per day; 400 mg per day was associated with toxicities.Serious adverse events included ECG QTc prolongation (*n* = 2), irreversible blurred vision (*n* = 1), and grade 3 nausea/vomiting (*n* = 3).^38^

Sensitivity to autophagy inhibition may be influenced by other tumor-specific factors including growth factor signaling, and recent preclinical data suggest that gliomas with either EGFRvIII or BRAFV600E mutations may be specifically sensitive.^39–41^ These data suggest that patient selection for specific biomarkers may play an important role in response rates and should be accounted for in future studies.

In conclusion, our trial, the largest of only 3 randomized trials in glioma and hydroxychloroquine/chloroquine adds useful information to the literature regarding the potential value of autophagy inhibition in glioma and other solid tumors. Our findings suggest that standard doses of hydroxychloroquine might not produce enhanced tumor regression with short-course radiotherapy. Investigators of similar trials being conducted should also carefully monitor PFS and OS. Results from ongoing and planned studies in glioma as well as other malignancies will further contribute to the growing evidence base for this type of therapy.

## Supplementary Material

vdaa046_suppl_Supplementary_Table_1

vdaa046_suppl_Supplementary_Table_2

vdaa046_suppl_Supplementary_Table_3

vdaa046_suppl_Supplementary_Table_4

vdaa046_suppl_Supplementary_Table_5

## References

[1] Philips A, Henshaw DL, Lamburn G, O’Carroll MJ Brain tumours: rise in glioblastoma multiforme incidence in England 1995-2015 suggests an adverse environmental or lifestyle factor. J Environ Public Health. 2018;2018:7910754. 30034480 10.1155/2018/7910754 PMC6035820

[2] Philips A, Henshaw DL, Lamburn G, O’Carroll MJ Authors’ comment on “Brain tumours: rise in glioblastoma multiforme incidence in england 1995-2015 suggests an adverse environmental or lifestyle factor”. J Environ Public Health. 2018;2018:2170208. 30046315 10.1155/2018/2170208 PMC6036793

[3] Roa W, Brasher PM, Bauman G, et al. Abbreviated course of radiation therapy in older patients with glioblastoma multiforme: a prospective randomized clinical trial. J Clin Oncol. 2004;22(9):1583–1588. 15051755 10.1200/JCO.2004.06.082

[4] Malmström A, Grønberg BH, Marosi C, et al.; Nordic Clinical Brain Tumour Study Group (NCBTSG) Temozolomide versus standard 6-week radiotherapy versus hypofractionated radiotherapy in patients older than 60 years with glioblastoma: the Nordic randomised, phase 3 trial. Lancet Oncol. 2012;13(9):916–926. 22877848 10.1016/S1470-2045(12)70265-6

[5] Minniti G, De Sanctis V, Muni R, et al. Hypofractionated radiotherapy followed by adjuvant chemotherapy with temozolomide in elderly patients with glioblastoma. J Neurooncol. 2009;91(1):95–100. 18758912 10.1007/s11060-008-9689-z

[6] Hegi ME, Diserens AC, Gorlia T, et al. MGMT gene silencing and benefit from temozolomide in glioblastoma. N Engl J Med. 2005;352(10):997–1003. 15758010 10.1056/NEJMoa043331

[7] Reifenberger G, Hentschel B, Felsberg J, et al.; German Glioma Network Predictive impact of MGMT promoter methylation in glioblastoma of the elderly. Int J Cancer. 2012;131(6):1342–1350. 22139906 10.1002/ijc.27385

[8] Perry JR, Laperriere N, O’Callaghan CJ, et al.; Trial Investigators Short-course radiation plus temozolomide in elderly patients with glioblastoma. N Engl J Med. 2017;376(11):1027–1037. 28296618 10.1056/NEJMoa1611977

[9] Weiner LM, Lotze MT Tumor-cell death, autophagy, and immunity. N Engl J Med. 2012;366(12):1156–1158. 22435376 10.1056/NEJMcibr1114526 PMC4389780

[10] Levy JMM, Towers CG, Thorburn A Targeting autophagy in cancer. Nat Rev Cancer. 2017;17(9):528–542. 28751651 10.1038/nrc.2017.53 PMC5975367

[11] Bishop E, Bradshaw TD Autophagy modulation: a prudent approach in cancer treatment? Cancer Chemother Pharmacol. 2018;82(6):913–922. 30182146 10.1007/s00280-018-3669-6 PMC6267659

[12] Poklepovic A, Gewirtz DA Outcome of early clinical trials of the combination of hydroxychloroquine with chemotherapy in cancer. Autophagy. 2014;10(8):1478–1480. 24991829 10.4161/auto.29428 PMC4203528

[13] Duffy A, Le J, Sausville E, Emadi A Autophagy modulation: a target for cancer treatment development. Cancer Chemother Pharmacol. 2015;75(3):439–447. 25422156 10.1007/s00280-014-2637-z

[14] Chude CI, Amaravadi RK Targeting autophagy in cancer: update on clinical trials and novel inhibitors. Int J Mol Sci. 2017;18(6): pii: E1279. 10.3390/ijms18061279 PMC5486101 28621712

[15] Weyerhäuser P, Kantelhardt SR, Kim EL Re-purposing chloroquine for glioblastoma: potential merits and confounding variables. Front Oncol. 2018;8:335. 30211116 10.3389/fonc.2018.00335 PMC6120043

[16] Sotelo J, Briceño E, López-González MA Adding chloroquine to conventional treatment for glioblastoma multiforme: a randomized, double-blind, placebo-controlled trial. Ann Intern Med. 2006;144(5):337–343. 16520474 10.7326/0003-4819-144-5-200603070-00008

[17] Briceño E, Reyes S, Sotelo J Therapy of glioblastoma multiforme improved by the antimutagenic chloroquine. Neurosurg Focus. 2003;14(2):e3. 10.3171/foc.2003.14.2.4 15727424

[18] Briceño E, Calderon A, Sotelo J Institutional experience with chloroquine as an adjuvant to the therapy for glioblastoma multiforme. Surg Neurol. 2007;67(4):388–391. 17350410 10.1016/j.surneu.2006.08.080

[19] Zarnett OJ, Sahgal A, Gosio J, et al. Treatment of elderly patients with glioblastoma: a systematic evidence-based analysis. JAMA Neurol. 2015;72(5):589–596. 25822375 10.1001/jamaneurol.2014.3739

[20] Kanzawa T, Kondo Y, Ito H, Kondo S, Germano I Induction of autophagic cell death in malignant glioma cells by arsenic trioxide. Cancer Res. 2003;63(9):2103–2108. 12727826

[21] Katayama M, Kawaguchi T, Berger MS, Pieper RO DNA damaging agent-induced autophagy produces a cytoprotective adenosine triphosphate surge in malignant glioma cells. Cell Death Differ. 2007;14(3):548–558. 16946731 10.1038/sj.cdd.4402030

[22] Golden EB, Cho HY, Jahanian A, et al. Chloroquine enhances temozolomide cytotoxicity in malignant gliomas by blocking autophagy. Neurosurg Focus. 2014;37(6):E12. 10.3171/2014.9.FOCUS14504 25434381

[23] Golden EB, Cho HY, Hofman FM, Louie SG, Schönthal AH, Chen TC Quinoline-based antimalarial drugs: a novel class of autophagy inhibitors. Neurosurg Focus. 2015;38(3):E12. 10.3171/2014.12.FOCUS14748 25727221

[24] Ye H, Chen M, Cao F, Huang H, Zhan R, Zheng X Chloroquine, an autophagy inhibitor, potentiates the radiosensitivity of glioma initiating cells by inhibiting autophagy and activating apoptosis. BMC Neurol. 2016;16(1):178. 27644442 10.1186/s12883-016-0700-6 PMC5029068

[25] Adamski V, Schmitt C, Ceynowa F, et al. Effects of sequentially applied single and combined temozolomide, hydroxychloroquine and AT101 treatment in a long-term stimulation glioblastoma in vitro model. J Cancer Res Clin Oncol. 2018;144(8):1475–1485. 29858681 10.1007/s00432-018-2680-y PMC11813370

[26] Rosenfeld MR, Ye X, Supko JG, et al. A phase I/II trial of hydroxychloroquine in conjunction with radiation therapy and concurrent and adjuvant temozolomide in patients with newly diagnosed glioblastoma multiforme. Autophagy. 2014;10(8):1359–1368. 24991840 10.4161/auto.28984 PMC4203513

[27] Rangwala R, Leone R, Chang YC, et al. Phase I trial of hydroxychloroquine with dose-intense temozolomide in patients with advanced solid tumors and melanoma. Autophagy. 2014;10(8):1369–1379. 24991839 10.4161/auto.29118 PMC4203514

[28] Rangwala R, Chang YC, Hu J, et al. Combined MTOR and autophagy inhibition: phase I trial of hydroxychloroquine and temsirolimus in patients with advanced solid tumors and melanoma. Autophagy. 2014;10(8):1391–1402. 24991838 10.4161/auto.29119 PMC4203516

[29] Mahalingam D, Mita M, Sarantopoulos J, et al. Combined autophagy and HDAC inhibition: a phase I safety, tolerability, pharmacokinetic, and pharmacodynamic analysis of hydroxychloroquine in combination with the HDAC inhibitor vorinostat in patients with advanced solid tumors. Autophagy. 2014;10(8):1403–1414. 24991835 10.4161/auto.29231 PMC4203517

[30] Patel S, Hurez V, Nawrocki ST, et al. Vorinostat and hydroxychloroquine improve immunity and inhibit autophagy in metastatic colorectal cancer. Oncotarget. 2016;7(37):59087–59097. 27463016 10.18632/oncotarget.10824 PMC5312297

[31] Chi KH, Ko HL, Yang KL, Lee CY, Chi MS, Kao SJ Addition of rapamycin and hydroxychloroquine to metronomic chemotherapy as a second line treatment results in high salvage rates for refractory metastatic solid tumors: a pilot safety and effectiveness analysis in a small patient cohort. Oncotarget. 2015;6(18):16735–16745. 25944689 10.18632/oncotarget.3793 PMC4599303

[32] Samaras P, Tusup M, Nguyen-Kim TDL, et al. Phase I study of a chloroquine-gemcitabine combination in patients with metastatic or unresectable pancreatic cancer. Cancer Chemother Pharmacol. 2017;80(5):1005–1012. 28980060 10.1007/s00280-017-3446-y

[33] Goldberg SB, Supko JG, Neal JW, et al. A phase I study of erlotinib and hydroxychloroquine in advanced non-small-cell lung cancer. J Thorac Oncol. 2012;7(10):1602–1608. 22878749 10.1097/JTO.0b013e318262de4a PMC3791327

[34] Vogl DT, Stadtmauer EA, Tan KS, et al. Combined autophagy and proteasome inhibition: a phase 1 trial of hydroxychloroquine and bortezomib in patients with relapsed/refractory myeloma. Autophagy. 2014;10(8):1380–1390. 24991834 10.4161/auto.29264 PMC4203515

[35] Eldredge HB, Denittis A, Duhadaway JB, et al. Concurrent whole brain radiotherapy and short-course chloroquine in patients with brain metastases: a pilot trial. J Radiat Oncol. 2013;2(3):315–321 10.1007/s13566-013-0111-x PMC3810973 24187608

[36] Rojas-Puentes LL, Gonzalez-Pinedo M, Crismatt A, et al. Phase II randomized, double-blind, placebo-controlled study of whole-brain irradiation with concomitant chloroquine for brain metastases. Radiat Oncol. 2013;8:209. 24010771 10.1186/1748-717X-8-209 PMC3848663

[37] Wen ZP, Zeng WJ, Chen YH, et al. Knockdown ATG4C inhibits gliomas progression and promotes temozolomide chemosensitivity by suppressing autophagic flux. J Exp Clin Cancer Res. 2019;38(1):298. 31291988 10.1186/s13046-019-1287-8 PMC6617611

[38] Compter I, Eekers D, Hoeben A, et al. CHLOROBRAIN phase IB trial: the addition of chloroquine, an autophagy inhibitor, to concurrent radiation and temozolomide for newly diagnosed glioblastoma. Ann Oncol. 2019; 30(suppl5):v154. 10.1080/15548627.2020.1816343 PMC8496728 32866424

[39] Jutten B, Keulers TG, Schaaf MB, et al. EGFR overexpressing cells and tumors are dependent on autophagy for growth and survival. Radiother Oncol. 2013;108(3):479–483. 23891088 10.1016/j.radonc.2013.06.033

[40] Jutten B, Keulers TG, Peeters HJM, et al. EGFRvIII expression triggers a metabolic dependency and therapeutic vulnerability sensitive to autophagy inhibition. Autophagy. 2018;14(2):283–295. 29377763 10.1080/15548627.2017.1409926 PMC5902239

[41] Levy JM, Thompson JC, Griesinger AM, et al. Autophagy inhibition improves chemosensitivity in BRAF(V600E) brain tumors. Cancer Discov. 2014;4(7):773–780. 24823863 10.1158/2159-8290.CD-14-0049 PMC4090283

