# Supplementary material for: Hydroxychloroquine and short-course radiotherapy in elderly patients with newly diagnosed high-grade glioma: a randomized phase II trial
Source: Neurooncol Adv. 2020 Apr 27;2(1):vdaa046. doi: 10.1093/noajnl/vdaa046 (PMC7236384; doi:10.1093/noajnl/vdaa046)
Supplement: vdaa046_suppl_Supplementary_Table_1 [file vdaa046_suppl_supplementary_table_1.docx]

**Supplementary Table 1. Cause of death**

| **Cause of Death** | **SCRT only**  **N=18** | **SCRT+HCQ**  **N=35** |
| --- | --- | --- |
| Disease progression | n (%)  11 (61.1) | 23 (65.7) |
|  |  |  |
| Lung Infection | 0 | 1 (2.9) |
|  |  |  |
| Pulmonary Embolism | 0 | 2 (5.7) |
|  |  |  |
| Myocardial ischaemia | 0 | 1 (2.9) |
|  |  |  |
| Bronchopneumonia | 0 | 1 (2.9) |
|  |  |  |
| Severe metabolic acidosis | 0 | 1 (2.9) |
|  |  |  |
| Disease related* | 1 (5.6) | 0 |
|  |  |  |
| Unknown | 5 (27.8) | 1 (2.9) |
|  |  |  |

*No evidence that the patient had progressed but the death was considered to be partly attributable to the presence of disease

Patients who had not died were censored at the date they were last known to be alive.

SCRT: short course radiotherapy

HCQ: hydroxychloroquine
