# Supplementary material for: Hydroxychloroquine and short-course radiotherapy in elderly patients with newly diagnosed high-grade glioma: a randomized phase II trial
Source: Neurooncol Adv. 2020 Apr 27;2(1):vdaa046. doi: 10.1093/noajnl/vdaa046 (PMC7236384; doi:10.1093/noajnl/vdaa046)
Supplement: vdaa046_suppl_Supplementary_Table_2 [file vdaa046_suppl_supplementary_table_2.docx]

**Supplementary Table 2. Adverse events of special interest for hydroxychloroquine**

| **Patients, %** | **SCRT only**  **N=18** | | **SCRT+HCQ**  **N=35** | |
| --- | --- | --- | --- | --- |
|  | **All grades** | **Grades ≥ 3** | **All**  **grades** | **Grades ≥ 3** |
|  |  |  |  |  |
| **Any ocular event** | **1 (5.6)** | **0** | **2 (5.7)** | **0** |
| Eye Disorders Other - Homonymous Hemianopia | 1 (**5.6**) | - | - | - |
| Floaters | - | - | 1 (2.9) | - |
| Weepy Eyes | - | - | 1 (2.9) | - |
|  |  |  |  |  |
| **Any change in bowel habits** | **6 (33.3)** | **2 (11.1)** | **16 (45.7)** | **2 (5.7)** |
| Acid Reflex/Heartburn | - | - | 1 (2.9) | - |
| Constipation | 2 (11.1) | 1 (5.6) | 3 (8.9) | - |
| Diarrhea | 2 (11.1) | - | 6 (17.1) | 1 (2.9) |
| Dry Mouth | - | - | 1 (2.9) | - |
| Flatulence | - | - | 1 (2.9) | - |
| Mouth Ulcers | 1 (**5.6**) | - | 1 (2.9) | - |
| Nausea | 2 (11.1) | 1 (5.6) | 11 (31.4) | 1 (2.9) |
| Oral Pain | - | - | 1 (2.9) | - |
| Rectal Haemorrhage | 1 (**5.6**) | - | - | - |
| Vomiting | 3 (16.7) | - | 4 (11.4) | - |
|  |  |  |  |  |
|  |  |  |  |  |
| **Any skin effects** | **6 (33.3)** | **-** | **15 (42.9)** | **1 (2.9)** |
| Alopecia | 6 (33.3) | - | 12 (34.3) | - |
| Erythema Multiform | 1 (**5.6**) | - | - | - |
| Pink Rash On Thighs | - | - | 1 (2.9) | - |
| Pruritis | 1 (**5.6**) | - | 1 (2.9) |  |
| Rash Maculopapular | - | - | 5 (14.3) | 1 (2.9) |
| Urticarial | 1 (**5.6**) | - | - | - |
|  |  |  |  |  |

SCRT: short course radiotherapy

HCQ: Hydroxychloroquine
