# Supplementary material for: Hydroxychloroquine and short-course radiotherapy in elderly patients with newly diagnosed high-grade glioma: a randomized phase II trial
Source: Neurooncol Adv. 2020 Apr 27;2(1):vdaa046. doi: 10.1093/noajnl/vdaa046 (PMC7236384; doi:10.1093/noajnl/vdaa046)
Supplement: vdaa046_suppl_Supplementary_Table_3 [file vdaa046_suppl_supplementary_table_3.docx]

**Supplementary Table 3: Grade 3-5 adverse events (n=7 for radiotherapy only vs n=21 for hydroxychloroquine group).**

| **Adverse event** | **SCRT only N=18** | **SCRT+HCQ n=35** |
| --- | --- | --- |
| Hip Fracture | 1 (5.6) | 0 |
| Fall | 1 (5.6) | 1 (2.9) |
| Fatigue | 1 (5.6) | 2 (5.7) |
| Cognitive Disturbance | 1 (5.6) | 0 |
| Seizure | 1 (5.6) | 1 (2.9) |
| Nausea | 1 (5.6) | 1 (2.9) |
| Constipation | 1 (5.6) | 0 |
| Depressed Level Of Consciousness | 0 | 1 (2.9) |
| Acute Coronary Syndrome # | 0 | 1 (2.9) |
| Alopecia | 0 | 1 (2.9) |
| Ankle Fracture | 0 | 1 (2.9) |
| Blurred Vision | 0 | 1 (2.9) |
| Confusion | 0 | 1 (2.9) |
| Diarrhea | 0 | 1 (2.9) |
| Dyspnea | 0 | 1 (2.9) |
| Lung Infection # | 0 | 5 (14.3) |
| Rash Maculopapular | 0 | 1 (2.9) |
| Steroid Induced Diabetes | 0 | 1 (2.9) |
| Thromboembolic Event | 0 | 1 (2.9) |

# Grade 5 adverse event (n=1 lung infection; n=1 acute coronary syndrome)
