# Supplementary material for: Hydroxychloroquine and short-course radiotherapy in elderly patients with newly diagnosed high-grade glioma: a randomized phase II trial
Source: Neurooncol Adv. 2020 Apr 27;2(1):vdaa046. doi: 10.1093/noajnl/vdaa046 (PMC7236384; doi:10.1093/noajnl/vdaa046)
Supplement: vdaa046_suppl_Supplementary_Table_4 [file vdaa046_suppl_supplementary_table_4.docx]

**Supplementary Table 4: Breakdown of the type of adverse events that led to discontinuation of trial drug in each of the 6 patients in the hydroxychloroquine arm.**

| **Event that led to HCQ discontinuation** | **Grade** |
| --- | --- |
| Intracranial Haemorrhage | 2 |
| Blurred vision | 3 |
| Blurred vision | 2 |
| Blurred vision | 2 |
| Rash maculopapular | 3 |
| Diarrhea | 3 |
