# Supplementary material for: Hydroxychloroquine and short-course radiotherapy in elderly patients with newly diagnosed high-grade glioma: a randomized phase II trial
Source: Neurooncol Adv. 2020 Apr 27;2(1):vdaa046. doi: 10.1093/noajnl/vdaa046 (PMC7236384; doi:10.1093/noajnl/vdaa046)
Supplement: vdaa046_suppl_Supplementary_Table_5 [file vdaa046_suppl_supplementary_table_5.docx]

**Supplementary Table 5. Quality of life data at 8 and 12 weeks compared to the baseline.**

|  | **HCQ arm**  **Median of: difference at 8 weeks - difference at baseline (N)** | **Control arm**  **Median of: difference at 8 weeks - difference at baseline (N)** | **P_value*** | **HCQ arm**  **Median difference at 12 weeks - Median difference at baseline (N)** | **Control arm Median difference at 12 weeks - Median difference at baseline (N)** | **P_value*** |
| --- | --- | --- | --- | --- | --- | --- |
| **Global health status / QoL** | 0 (17) | 0 (10) | 0.63 | 0 (13) | -8.3 (7) | 1 |
| **Physical Functioning** | -13.3 (19) | -13.3 (11) | 0.59 | -10.0(14) | -20.0(8) | 0.51 |
| **Emotional Functioning** | 0 (19) | 0 (11) | 0.73 | -9.7(10) | -20.8(6) | 0.40 |
| **Cognitive Functioning** | 0 (19) | 0(11) | 0.84 | -16.7 (13) | 0 (8) | 0.66 |
| **Social Functioning** | -16.7 (19) | 0 (11) | 0.68 | -0(13) | -8.3(8) | 0.71 |
| **Fatigue** | 11.1 (19) | 0 (11) | 0.50 | 7.9 (14) | 20.8 (8) | 0.25 |
| **Nausea / Vomiting** | 0 (19) | 0 (11) | 0.22 | 0 (13) | 0 (8) | 0.49 |
| **Pain** | 0 (19) | 0 (11) | 0.74 | 0 (14) | 0 (8) | 0.51 |
| **Dyspnoea** | 0 (17) | 0 (11) | 0.62 | 0 (12) | 0 (8) | 0.89 |
| **Insomnia** | 0 (19) | 0 (11) | 0.25 | 0 (14) | -16.7 (8) | 0.21 |
| **Appetite loss** | 0(19) | 0(11) | 0.02** | 0 (14) | 0 (8) | 0.55 |
| **Constipation** | 0 (18) | 0 (11) | 0.48 | 0 (12) | 0 (8) | 0.51 |
| **Diarrhoea** | 0 (18) | 0 (11) | 0.35 | 0(12) | 0(8) | 0.65 |
| **Financial Problems** | 0 (19) | 0 (10) | 0.60 | 0 (13) | 0 (7) | 0.26 |

*Two sided p_value for the Non parametric Wilcoxon Mann Whitney to test the difference between arms.

** We should bear in mind that number of patients are very low so we would not have the power to detect small differences.
